# Membrane‐Mediated Force Transduction Drives Stick‐Slip Motion of Lipid Vesicles

**DOI:** 10.1002/advs.202517219

**Published:** 2026-01-26

**Authors:** Paula Magrinya, Arin Escobar Ortiz, Juan L. Aragones, Laura R. Arriaga

**Affiliations:** ^1^ Department of Theoretical Condensed Matter Physics Universidad Autónoma de Madrid Madrid 28049 Spain; ^2^ Condensed Matter Physics Center (IFIMAC) Universidad Autónoma de Madrid Madrid 28049 Spain; ^3^ Instituto de Ciencia de Materiales Nicolás Cabrera Universidad Autónoma de Madrid Madrid 28049 Spain

**Keywords:** driven matter, lubrication, membranes, vesicles

## Abstract

How internal forces are transduced into motion through soft, fluid membranes remains a fundamental question in the study of active systems. This is investigated using a minimal system: a ferromagnetic particle encapsulated inside a lipid vesicle with controlled membrane composition and phase behavior. A rotating magnetic field drives particle rotation, generating internal flow. This flow propels the particle along the inner membrane leaflet and induces local membrane slip, with regions closer to the particle exhibiting faster motion relative to the substrate. When the particle approaches the vesicle bottom, this slip produces a shear gradient across the lubrication gap, resulting in vesicle propulsion through a stick‐slip cycle. Vesicle motion depends on membrane elasticity, excess area, and phase coexistence. Deformations and fluctuations dissipate stress, while line tension deflects the particle and reorients membrane structure. The results demonstrate how lipid membranes mediate force transduction and motion, offering new avenues for the bottom‐up design of soft, membrane‐based active systems.

## Graphical abstract

A rotating magnetic field actuates a ferromagnetic particle inside a lipid vesicle, generating internal flows that induce stick‐slip propulsion near a surface. Membrane properties—elasticity, excess area, and phase behavior—modulate this motion by shaping stress dissipation and force transduction. The system reveals how soft membranes regulate active transport and inspire membrane‐based micromachine design.

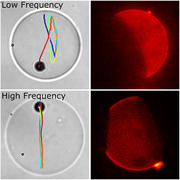

## 1 Introduction

The ability of cells to convert internal active forces into shape deformations and directed motion^[^ ^1^ ^]^ is central to diverse processes such as cell migration, endocytosis and mechanosensing.^[^ ^2^, ^3^, ^4^, ^5^ ^]^ This force transduction occurs through the lipid membrane, a soft and fluid interface that plays an active mechanical role. Unlike solid boundaries, fluid membranes deform elastically in response to external or internal forces,^[^ ^6^, ^7^, ^8^, ^9^, ^10^ ^]^ or due to spontaneous thermal undulations—responses governed by membrane tension and bending rigidity.^[^ ^11^ ^]^ Phase coexistence further enriches their mechanics by introducing line tension at domain boundaries,^[^ ^12^ ^]^ while membrane viscosity allows flow^[^ ^13^, ^14^ ^]^ and shear dissipation.^[^ ^15^ ^]^ As a result, fluid membranes can redirect and transmit stresses tangentially despite lacking shear elasticity, making them active regulators of force transmission. As a minimal, controllable model system, giant unilamellar vesicles (GUVs) provide a platform to study the membrane‐mediated force transduction. When subjected to external shear or Poiseuille flows, the lipid membrane of GUVs exhibits characteristic deformations and tank‐treading,^[^ ^16^, ^17^, ^18^, ^19^, ^20^, ^21^, ^22^, ^23^ ^]^ reorganizes membrane domains^[^ ^18^, ^19^ ^]^ and transduces external shear stresses into internal flows.^[^ ^15^, ^20^ ^]^ In contrast, how internally generated forces—such as cytoplasmic streaming driven by cortical motors in living cells^[^ ^24^, ^25^, ^26^, ^27^, ^28^ ^]^—contribute to force transduction and motion is less understood. Though often associated solely with intracellular mixing,^[^ ^29^ ^]^ these internal flows likely play a more complex role in shaping membrane dynamics and motion. Addressing this open question requires synthetic systems with controllable internal activity. While theoretical models examine how internal rotating particles generate flows in confined geometries,^[^ ^30^, ^31^, ^32^ ^]^ and recent experiments with magnetic particles encapsulated in solid‐like polymer vesicles demonstrate efficient force transmission through rigid membranes,^[^ ^33^ ^]^ the coupling between internal activity and fluid lipid membranes remains largely unexplored.^[^ ^34^ ^]^

**Figure 1:**
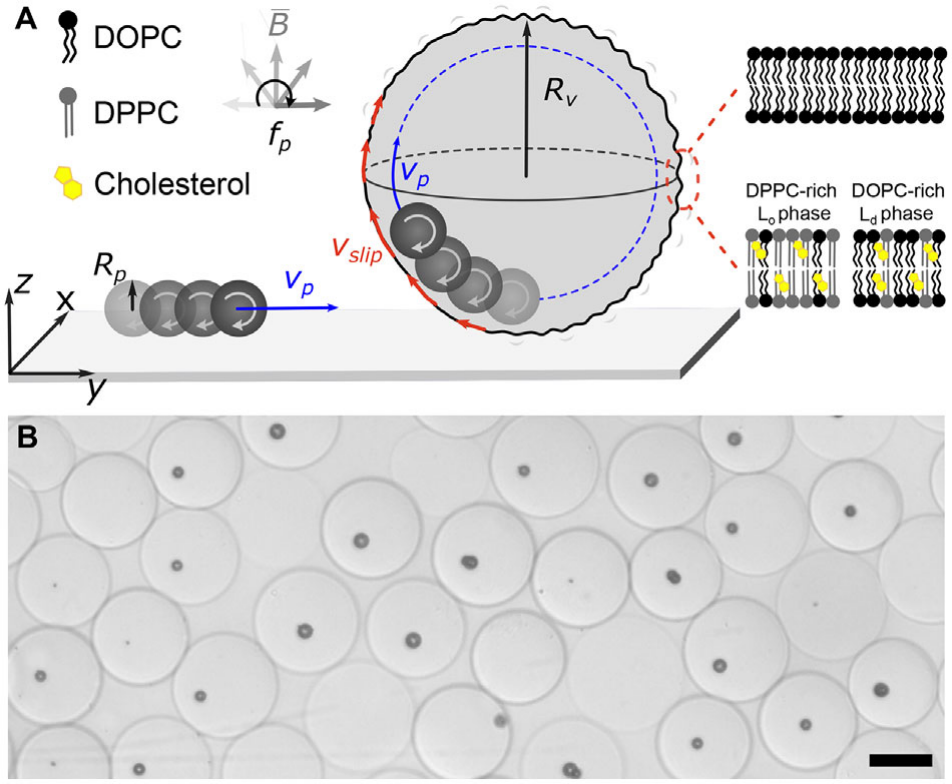
Experimental system. A) Schematic illustration. A single ferromagnetic particle is rotated about the *x*‐axis at frequency *f*_*p*_. Free and confined particles rotate at a frequency *f*_*p*_, around the *x*‐axis. When free, the particle translates along the positive *y*‐axis driven by the shear stress across the lubrication gap. When confined within a vesicle, it performs circular loops in the vesicle equator (*yz*‐plane) with opposite sense of translation becuase membrane curvature enhances the pressure‐driven component of motion; the rotating particle imposes a local membrane slip velocity *v*_*slip*_ and deforms the nearby membrane. The membrane can be composed of either pure DOPC or a ternary mixture of DOPC/DPPC/cholesterol, which separates into liquid disordered (*L*_*d*_) and liquid ordered (*L*_*o*_) phases with initial Janus morphology. B) Representative optical microscope image of DOPC‐GUVs, fabricated by microfluidics, encapsulating a single particle (black appearance) or none. Scale bar is 50 µm.

Here, to investigate how fluid membranes mediate the transduction of internal forces into whole‐vesicle motion, we develop a minimal system consisting of a single ferromagnetic particle encapsulated within a lipid GUV, as illustrated schematically in **Figure** **1**A. The particle is actuated by an external rotating magnetic field, generating internal fluid flows that interact with the fluid vesicle membrane. This setup allows us to examine not only how active stresses are transmitted to the membrane and drive motion, but also how the membrane mechanical properties—including fluidity, excess area, and compositional heterogeneity—modulate, dissipate or redirect these stresses, revealing a dynamic interplay between internal activity and membrane response.

## 2 Results and Discussion

### 2.1 Rotating Microparticles Confined in Vesicles

To form vesicles with controlled size and membrane composition, each encapsulating a single ferromagnetic particle, we use water‐in‐oil‐in‐water (W/O/W) double emulsion drops with ultrathin oil shells as templates.^[^ ^35^ ^]^ These templates are produced with a co‐flow glass‐capillary microfluidic device.^[^ ^36^ ^]^ An overview micrograph of representative vesicles produced by microfluidics is shown in Figure 1B. We study two different membrane compositions that produce fluid‐like membranes, 100 mol% 1,2‐dioleoyl‐sn‐glycero‐3‐phosphocholine (DOPC) and a mixture containing 37.4 mol% DOPC, 37.4 mol% dipalmitoyl‐sn‐glycero‐3‐phosphocholine (DPPC), 25 mol% cholesterol (Chol) and 0.2 mol% rhodamine B 1,2‐dihexadecanoyl‐sn‐glycero‐3‐phosphoethanolamine (DHPE‐Rh), the later exhibiting liquid disordered (L_d_) ‐ liquid ordered (L_o_) phase coexistence,^[^ ^37^ ^]^ with the L_d_ phase fluorescently labeled. The inner core of the vesicles contains 2 wt.% poly(vinyl alcohol) (PVA, 13 000–23 000 g mol^−1^) and 8 wt.% poly(ethylene glycol) (PEG, 6000 g mol^−1^). PEG primarily raises the bulk viscosity of the encapsulated aqueous phase. It is not necessary for GUV assembly in our microfluidic method and can be substituted by water or by a sucrose solution; we use PEG‐free formulations, in which PEG is replaced by a 200 mM sucrose solution, to produce the smallest vesicles, because the increased viscosity of PEG limits droplet breakup in the microfluidic device. By contrast, PVA may adsorb to and weakly insert into the bilayer, thereby increasing membrane surface viscosity and tension. These anticipated changes may slow down membrane dynamics relative to PVA‐free vesicles. The external medium consists of an aqueous solution of either sucrose or glucose, which matches the osmolarity of the vesicle lumen to prevent mechanical stresses on the lipid membrane, simultaneously enabling vesicle sedimentation based on the density differences between the inner and external media. For observation, vesicles are transferred to a chamber with the bottom coated with bovine serum albumin (BSA) to prevent vesicle adhesion.

To drive particle rotation, we use an external rotating magnetic field of 10 mT with rotation axis parallel to the substrate (*x*‐axis, Figure 1A) and rotation frequency, *f*_*p*_, varying from 1 to 10 Hz. Particle rotation creates a rotational flow in the fluid where it is suspended. In the lubrication limit, the presence of a limiting surface (the substrate) breaks the symmetry of the rotational flow, coupling the rotational and translational degrees of freedom of the particle. On the planar substrate, the no slip‐boundary condition breaks the top‐down symmetry of the flow field, creating a shear force that makes a clockwise rotating particle translate along the positive *y*‐axis or rolling direction,^[^ ^38^, ^39^, ^40^, ^41^ ^]^ as illustrated schematically in the leftmost part of Figure 1A. Importantly, this roto‐translational coupling is directly influenced by the geometry of the limiting surface.^[^ ^42^, ^43^, ^44^ ^]^ On a curved substrate, on the inner leaflet of the vesicle membrane, the particle follows circular loops along the vesicle equator (*yz*‐plane) moving in the direction opposite to that of the free particle, as illustrated in Figure 1A and Movie S1 (Supporting Information). This sliding motion occurs due to the increased pressure gradient that results from the breaking of the fore‐aft symmetry of the rotational flow field on the curved limiting membrane.^[^ ^33^, ^34^, ^45^, ^46^ ^]^ The particle thus slides along the vesicle equator until a thermal or mechanical perturbation seeds a slow drift; it then follows a spiral trajectory toward one of the vesicle poles (+*x* or −*x*), guided by the presence of a Hill vortex perpendicular to the particle circular trajectory, as previously reported in vesicles with solid‐like membranes.^[^ ^33^ ^]^ The formation of the Hill vortex comes from the polar components of the fluid flow resulting from the curvature of the confining surface and therefore, a similar drift can occur in fluid vesicles.

### 2.2 Flow Transmission Across Fluid Membranes

**Figure 2:**
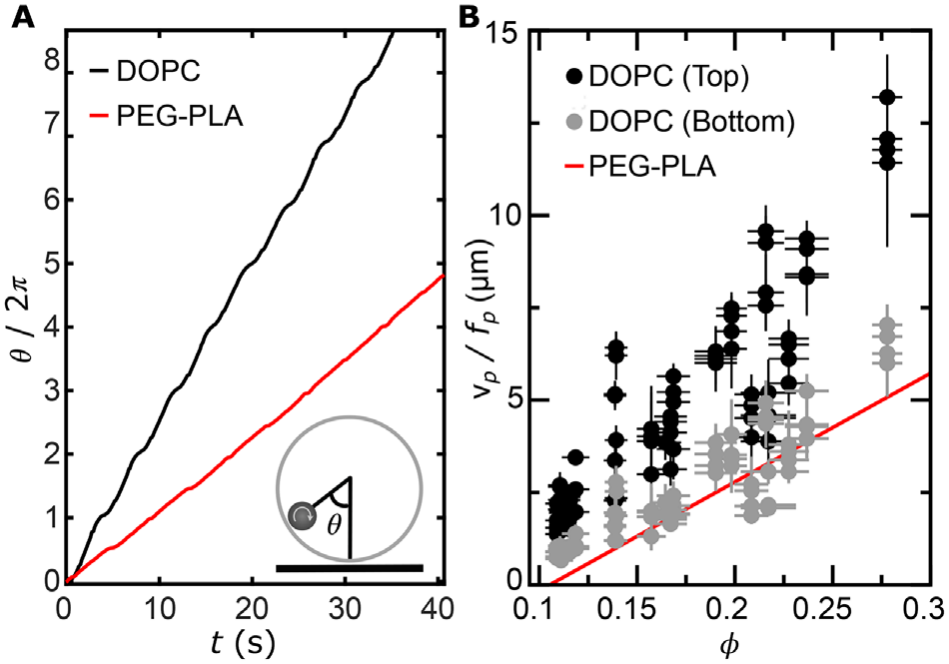
Rotating particle confined within fluid vesicles. A) Representative particle angular trajectory θ(*t*) within a fluid (DOPC) or a solid‐like (PEG‐PLA) vesicle for a particle rotating at frequency *f*_*p*_ = 10 Hz and degree of confinement ϕ = 0.15. The instantaneous angular velocity is *d*θ/*dt*; hence flatter (steeper) segments correspond to lower (higher) sliding velocities. The sliding velocity is computed as *v*_*p*_(*t*) = *R*_*V*_ *d*θ/*dt*, yielding minimum *v*_*p*_ near the bottom and maximum *v*_*p*_ near the top, with periodic modulation. B) Distance traveled by the particle per full rotation, *v*_*p*_/*f*_*p*_, along its circular loops as a function of the degree of confinement, ϕ, measured when the particle is near the top or bottom of DOPC vesicles. Data are reported as mean ± SD across vesicles (n = 19); each datum is the average over 5 cycles per vesicle. The red line is the case of PEG‐PLA vesicles.^[^ ^33^ ^]^

To determine the sliding velocity, *v*_*p*_, of the particle on the inner leaflet of the lipid membrane, we track its angular position θ(*t*) in bright‐field microscope images along its circular path, considering θ = 0 at the vesicle bottom. The instantaneous angular velocity ω is the slope *d*θ/*dt* and thus *v*_*p*_ = *R*_*v*_ω, where *R*_*v*_ is the vesicle radius. Interestingly, in stark contrast to polyethylene glycol‐polylactic acid (PEG‐PLA) polymer vesicles with solid‐like membranes,^[^ ^33^ ^]^ we observe that θ(*t*) displays periodically alternating flat and steep segments, as exemplified in **Figure** **2**A. This indicates that in each loop, the particle slows down as it approaches the bottom of the vesicle, reaching its minimum velocity at this position. It then increases its velocity, reaching the maximum value at the top of the vesicle, repeating this cycle in successive loops.

To understand this periodic modulation, we complementarily characterize the effect of confinement, defined as ϕ = *R*_*p*_/*R*_*v*_, on the sliding velocity of the particle at the top and bottom of the vesicle and normalize them by the particle rotational frequency. This normalization yields the distance traveled by the particle along the membrane per full rotation. Remarkably, at the bottom of the vesicle, this distance increases linearly with ϕ and is slightly larger than the values reported for solid‐like vesicles,^[^ ^33^ ^]^ as shown by the gray symbols lying just above the red line in Figure 2B. Moreover, at the top of the vesicle, the distance traveled per rotation is consistently larger than both at the bottom and in the solid case, with a noticeable steeper linear increase with ϕ, as shown by the black symbols in Figure 2B. This distinct particle dynamics at the top and bottom of the vesicle suggests that the interplay between shear and pressure varies along the particle trajectory. In contrast to a solid boundary, a fluid lipid bilayer allows for flow transmission across the bilayer.^[^ ^15^, ^20^ ^]^ At the top of the vesicle, where the outer fluid is unbounded, the flow generated by particle rotation dissipates, resulting in minimal shear stress and a higher sliding velocity. However, as the particle approaches the vesicle bottom, the flow transmitted across the membrane encounters the solid substrate beneath the vesicle. This additional hydrodynamic boundary enhances the local shear stress acting on the particle, thereby reducing its sliding velocity.^[^ ^30^, ^31^ ^]^ However, the local shear stress on the particle at the bottom of fluid‐like vesicles remains smaller than in solid‐like vesicles,^[^ ^33^ ^]^ because in the fluid case, the substrate lies beneath the membrane, whereas in the solid‐like case, the rigid membrane itself acts as the substrate. This additional separation in the fluid vesicle reduces the shear transmission to the particle, allowing pressure gradients to play a more predominant role in the dynamics of the rotating particle.

### 2.3 Interplay Between Membrane Shape Fluctuations and Particle Dynamics

**Figure 3:**
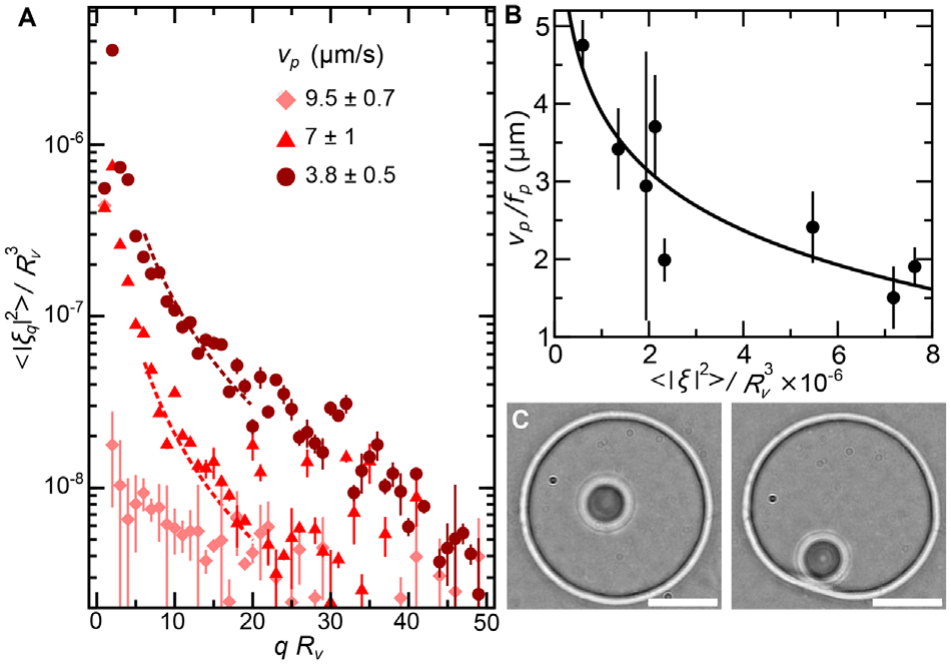
Interplay between shape fluctuations and particle dynamics. A) Representative variation of the fluctuation amplitude, 〈|ξ_*q*_|^2^〉, as a function of the wavevector *q* for a vesicle of radius, *R*_*v*_, that undergoes osmotic deflation. Data are presented as mean ± SD over >2000 contours. Dashed lines correspond to fits using the Helfrich spectrum (Equation 4), yielding a bending modulus κ_*b*_ = 7.6 *k*_*B*_ *T*, and membrane tensions σ = 2.57 × 10^−6^ N·m^−1^(triangles) and σ = 1.14 × 10^−6^ N·m^−1^ (circles). The legend shows the particle sliding velocity measured at each tension state. B) Variation of the distance traveled by the confined particle per full rotation as a function of the fluctuation amplitude. Data are presented as mean ± SD across n=4 vesicles, with >2000 contours per vesicle. The black line is a fit to a logarithmic dependence of *v*_*p*_/*f*_*p*_∝log (1/〈|ξ|^2^〉). C) Representative bright field microscope images showing visible shape deformations in a deflated vesicle as the particle approaches the vesicle membrane. Scale bar 25μm.

To understand how membrane mechanics influences particle dynamics, we examine the interaction between the encapsulated particle—sliding along the inner membrane leaflet— and the vesicle membrane under varying tension conditions. Specifically, we induce excess membrane area in the vesicles by allowing evaporation of the outer medium, which concentrates the external solution and causes the vesicle to deflate. This deflation leads to a reduction in membrane tension and the emergence of shape fluctuations, providing a platform to probe their impact on particle sliding. To characterize membrane shape fluctuations at different deflation states, we perform flickering spectroscopy, which quantifies thermally driven shape fluctuations of GUVs by tracking their midplane contour in time and decomposing it into Fourier modes; the fluctuation spectrum 〈|ξ_*q*_|^2^〉 is then fit to the standard Helfrich model to extract membrane tension and bending rigidity. Once the fluctuation spectra for each tension state is recorded, we apply the magnetic field to drive particle rotation and measure its sliding velocity at fixed *f*_*p*_ and ϕ. The particle sliding velocity decreases by up to 40% with increasing fluctuation amplitude, as shown in **Figure** **3**A, where each curve is color‐coded according to its associated particle velocity to provide a direct correlation between fluctuation amplitude and sliding behavior.

To further explore the link between membrane fluctuations and sliding dynamics, we examine how the fluctuation amplitude 〈|ξ|^2^〉 = ∑_*q*_〈|ξ_*q*_|^2^〉 correlates with the distance traveled by the particle in each full rotation, *v*_*p*_/*f*_*p*_. By analyzing a range of vesicles with varying tension states, we find that this distance systematically decreases as fluctuation amplitudes increase, as shown in Figure 3B. This trend is consistent with the particle moving closer to the inner membrane leaflet for smaller fluctuation amplitudes, where viscous coupling between particle rotation and translation becomes more effective. In contrast, large membrane fluctuations generate a repulsive pressure that increases the separation distance between the particle and the membrane, thereby weakening the hydrodynamic coupling.^[^ ^30^ ^]^ This repulsion, known as the undulation pressure, arises from the entropic confinement of the shape fluctuations of a membrane as it approaches another surface and follows a power‐law dependence with the distance to the confining substrate *P*(*d*) ≈ (*k*_*B*_ *T*)^2^/κ_*b*_ *d* ^3^,^[^ ^11^ ^]^ where *k*_*B*_ is the Boltzmann constant, *T* is the temperature, κ_*b*_ is the membrane bending modulus, and *d* is the separation distance between the inner membrane leaflet and the surface of the particle in our particular case. Considering that this distance is set by the fluctuation amplitude, *d*∝〈|ξ|^2^〉^1/3^, we find the expected logarithmic dependence between particle velocity and particle‐membrane separation distance,^[^ ^38^, ^46^ ^]^ as shown by the black line in Figure 3B.

To explore how the motion of the particle within the vesicles influences membrane shape, we analyze membrane deformations during particle sliding and find that the membrane becomes visibly disturbed, specially when the excess area is sufficiently large, as shown in Figure 3C. Notably, the fluctuation spectrum is only perturbed at long wavelengths, *qR*_*v*_ < 4 (Figure S1, Supporting Information), suggesting that particle dynamics modulates the apparent membrane tension, without affecting membrane bending rigidity. In this tension‐dominated regime, the membrane responds dynamically to the active hydrodynamic stresses imposed by the particle, redistributing its excess area through local shape deformations. This dynamic reshaping of the vesicle under internal hydrodynamic forcing bears resemblance to the cell membrane deformations driven by actomyosin activity, where internally generated active stresses modulate membrane tension and redistribute excess area to produce shape changes.^[^ ^47^ ^]^

### 2.4 Interplay Between Membrane Phase Organization and Particle Motion

To further investigate the reciprocal coupling between membrane mechanics and particle dynamics, we study vesicles with phase coexistence, initially exhibiting a Janus configuration with two distinct membrane domains that differ in their mechanical properties. In contrast to single‐phase membranes, the presence of coexisting domains introduces spatial variations in mechanical properties that may further modulate the local dynamics of the encapsulated particle. Interestingly, however, we find the same dependence of *v*_*p*_/*f*_*p*_ on ϕ as in single fluid membranes (Figure S2, Supporting Information), suggesting that differences in membrane mechanics between L_d_ and L_o_ domains play a minor role. Importantly, we observe off‐trajectory displacements as particles encounter the domain boundary (Movie S2, Supporting Information), which points to line tension as a key factor influencing particle dynamics in phase‐separated membranes. This effect is most pronounced at low rotational frequencies (*f*_*p*_ ⩽ 2 Hz), where the particle remains confined to a single domain and its motion is visibly constrained by the membrane phase organization.

**Figure 4:**
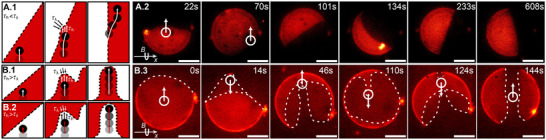
Membrane phase organization. Schematic illustration (left) and representative epi‐fluorescence microscope images (right) of a phase‐separated vesicle, with the L_d_ phase labeled in red; and the encapsulated particle, slightly visible as a dark spot highlighted as a white circle for visualization purposes. Particle rotating around the *x*‐axis induces: A) domain alignment perpendicular to the *x*‐axis when *f*_*p*_ = 2 Hz (τ_*h*_ < τ_λ_) or B) a one‐phase belt at the equator when *f*_*p*_ = 5 Hz (τ_*h*_ > τ_λ_). Scale bars are 25μm.

Line tension, the interfacial energy per unit length at the boundary between coexisting domains, arises from a local mismatch in membrane packing and does not act as a force on its own. However, when the sliding particle generates a local shear flow in the membrane, the domain boundary resists being deformed. The viscous stress induced by the sliding particle scales as τ_*h*_ ≈ η_*m*_ *v*_*slip*_/*R*_*p*_ ≈ η_*m*_2π*f*_*p*_, while the line tension restoring stress that tends to avoid deformation of a boundary of radius *R*_*p*_ scales as τ_λ_ ≈ λ/*R*_*p*_. The balance between these two stresses defines the capillary number C*a* = η_*m*_ *v*_*slip*_/λ. Over an arc of length ≈*R*_*p*_, the imbalance (τ_*h*_ − τ_λ_) generates an in‐plane torque $M\approx \left(\tau_{h}-\tau_{\lambda }\right)R_{p}^{2}$ that rotates the domain boundary toward the equator. Consistent with this local stress‐torque picture, at low *f*_*p*_, we observe boundary reorientation, as shown in **Figure** **4**A and Movie S3 (Supporting Information), indicating that τ_*h*_ < τ_λ_ (C*a* < 1).

**Figure 5:**
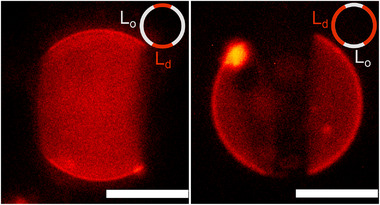
Three‐domain configuration. Epi‐fluorescence microscope image of two representative GUVs showing that the one‐phase belt at the equator may be either L_d_ (left) or L_o_ (right). Scale bar 25μm.

At higher rotational frequencies (*f*_*p*_ > 2 Hz), the balance reverses (τ_*h*_ > τ_λ_, as τ_*h*_ grows linearly with *f*_*p*_) and the particle begins to mechanically remodel the membrane phase structure (C*a* > 1). Using our scaling, the crossover frequency is *f*_*c*_ ≈ λ/(2πη_*m*_ *R*_*p*_). Above this threshold, the initial Janus configuration is progressively transformed into a three‐domain structure, stabilized by the continuous sliding of the particle along the vesicle equatorial plane, as shown in Figure 4B and Movie S4 and S5 (Supporting Information). There is no compositional exchange between domains but a mechanical deformation that results in domain splitting. The split domain can be either L_o_ or L_d_, as shown in **Figure** **5** (Movies S4 and S5, Supporting Information), which supports the view that line tension, rather than the specific mechanical contrast between domains, governs reorganization under strong driving. Consistent with this interpretation, we observe the same alignment‐remodeling transition and three‐domain state in vesicles prepared by electroformation without PVA or other polymers, confirming that polymer‐membrane interactions or heterogeneous decorations of membrane regions by polymers are not responsible for the observed behavior, as shown in Figure S3 (Supporting Information).

To show the generality of the behavior illustrated by Figure 4, we provide a phase organization diagram showing the behavior of the domains—either aligning or forming a one‐phase equatorial road— as a function of *f*_*p*_ and ϕ, performed on 70 vesicles, in Figure S4 (Supporting Information). In addition, we perform experiments using an alternative actuation mode where we apply the magnetic field around the *z*‐axis, perpendicular to the substrate, making the particle spin in place at the vesicle bottom. In this configuration, for low frequencies (*f*_*p*_ ⩽ 2 Hz), the domain boundary aligns perpendicular to the axis of rotation as in the sliding case (Figure S5A, Supporting Information). In contrast, when increasing the particle spinning frequency, the particle initially rotates directly at the domain boundary, gradually deforming it until the interface closes in on itself, creating the same type of belt observed in the sliding case. However, unlike in the sliding mode, the particle remains spinning outside the belt, confined within the small domain created, (Figure S5B, Supporting Information). These complementary observations reinforce that membrane‐phase reorganization can be robustly driven by hydrodynamic activity alone, independent of the specific mode of particle actuation.

The emergence of a three domain configuration provides two‐domain boundaries that effectively stabilize the circular trajectory of the particle along the vesicle equator. In single‐phase vesicles, wether fluid or solid‐like, the particle typically deviates from the equatorial plane and spirals toward one of the vesicle poles (See Movie S1, Supporting Information). In solid‐like membranes, this behavior is attributed to the formation of an asymmetric Hill vortex that exerts a tangential force on the particle until the vortex dissipates.^[^ ^33^ ^]^ While the drift velocity toward the pole, *v*_*pole*_, remains comparable between fluid and solid‐like membranes (Figure S6, Supporting Information), the likelihood of reaching the pole is significantly reduced in fluid‐like membranes: only 35% in DOPC vesicles compared to 70% in PEG‐PLA vesicles. In most cases, the particles confined in fluid‐like membranes follow spiraling trajectories that settle into steady‐state orbits at intermediate latitudes (Movie S6, Supporting Information). This reduced poleward displacement likely reflects a different internal flow structure within fluid vesicles. In contrast, the introduction of two domain boundaries in membranes with fluid coexistence provides line‐tension‐mediated restoring stresses that couple hydrodynamically to the sliding particle and limit off‐equatorial drift, thereby stabilizing the equatorial trajectory.

### 2.5 Asymmetric Membrane Slip and Stick‐Slip Vesicle Translation

**Figure 6:**
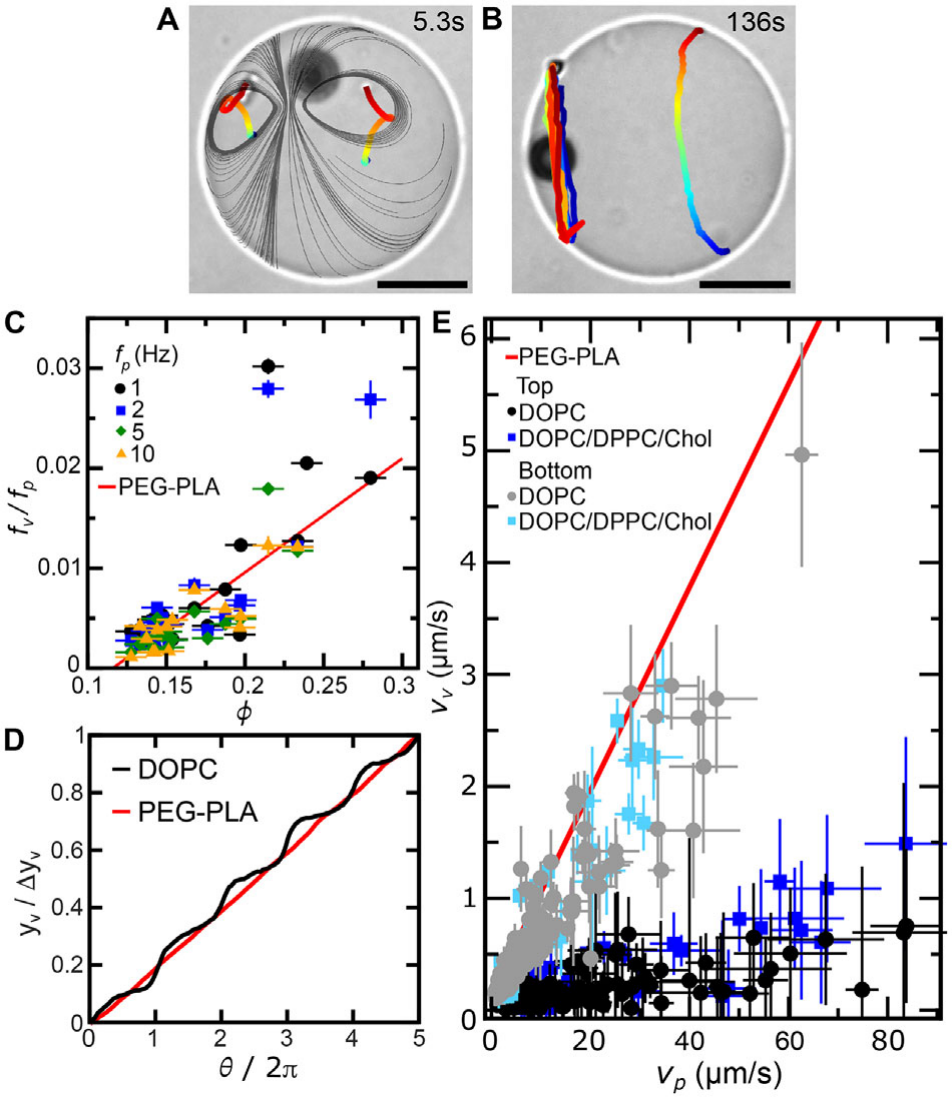
Stick‐slip vesicle motion. A,B) Trajectories of two membrane defects when the encapsulated particles is (A) at the equator or (B) at the pole. Gray lines in (A) correspond to the theoretical streamlines expected for a point force on the membrane.^[^ ^14^ ^]^ Scale bar 25μm. C) Rotation frequency of the membrane at the equatorial region as a function of the degree of confinement for phase‐separated vesicles. Data is presented as mean ± SD with n=20. D) Evolution of the vesicle center of mass as a function of the angular position of the particle within the vesicle for both PEG‐PLA and DOPC membranes with ϕ = 0.24 and *f*_*p*_ = 5Hz. E) Translational velocity of vesicles as a function of particle velocity and position within the vesicle (top, bottom) for DOPC, and DOPC/DPPC/Chol membranes. Data is presented as mean ± SD with n=71. The red line corresponds to solid‐like PEG‐PLA vesicles.^[^ ^33^ ^]^

To explore how internal flow couples to membrane motion, we track membrane defects during particle sliding. When the particle slides along the vesicle equator, membrane defects located symmetrically with respect to the equatorial plane trace vortex‐like trajectories, as shown in **Figure** **6**A and Movie S7 (Supporting Information). This is consistent with the dipolar flow field expected from a tangential point force applied to the membrane (Figure S7).^[^ ^13^, ^14^ ^]^ By contrast, when the particle rotates at the vesicle pole, membrane defects trace circular trajectories parallel to the equator, as shown in Figure 6B and Movie S7 (Supporting Information), with a rotation frequency that decreases with increasing defect‐particle distance. This behavior is consistent with the streamline patterns generated by a point torque applied to the membrane, which produces concentric circular flows around the axis of rotation, with angular velocity decreasing with radial distance.^[^ ^13^, ^14^ ^]^ These observations confirm that membrane motion in vesicles with fluid membranes arises from local membrane flow rather than from rigid‐body rotation. In fact, rigid‐body rotation is only expected when the vesicle radius is smaller than the Saffman length, *L*_*s*_ = η_*m*_/2η, which defines the length scale beyond which momentum dissipation into the surrounding fluid dominates over viscous shear within the membrane.^[^ ^13^, ^14^ ^]^

To characterize the effect of confinement in the coupling between internal and membrane flows, we measure the rotational frequency of the vesicle, *f*_*v*_, at the equatorial plane, by tracking the domain boundary in Janus vesicles (Figure S5, Supporting Information), while driving particle spinning around the the *z*‐axis at the vesicle bottom. Unlike tracking of membrane defects during particle sliding, this method allows us to probe the membrane flow field at a controlled radial distance from the point of torque application. The rotation frequency obtained by this method, and its dependence on ϕ, closely resembles that of vesicles with solid‐like membranes,^[^ ^33^ ^]^ as shown in Figure 6C. This similarity reflects that the net torque is comparable in both cases.

To investigate how fluid membranes mediate force transduction during vesicle translation along the substrate, we track the center of mass of the vesicle along the positive *y*‐axis. We observe that vesicle displacements are more pronounced when the sliding particle occupies the bottom region of the vesicle compared to when it occupies the top region, as shown by the black line in Figure 6D. The origin of this stick‐slip motion lies in the inner flow structure and viscous dissipation within a fluid membrane. Because the flow generated by particle rotation applies a point force on the closest membrane region that decays as 1/*r* (Figure S7, Supporting Information), the local membrane velocity $v_{slip}^{bottom}$ when the particle is at the top must be smaller than that when the particle is at the bottom. In addition, since there is not rigid‐body membrane rotation, the circulation of the fluid velocity field along any inner closed loop over the vesicle surface must be zero, and thus, the net vorticity of the inner flows should vanish. This constraint results in the formation of an internal counterrotating vortex at the vesicle bottom when the particle is at the top, which opposes the field generated by the point force applied by the rotating particle, resulting in a nearly zero slip velocity in the membrane region near the substrate ($v_{\text{slip}}^{\text{bottom}}\approx 0$). Consequently, when the particle rotates at the top of the vesicle, there is negligible shear stress across the lubrication gap between the vesicle and the substrate, thereby producing no net vesicle translation (stick). In good agreement with this scenario, we measure a very small *v*_*v*_ in single‐phase and two‐phase fluid vesicles independently on particle sliding velocity, as shown by the black circles and blue squares in Figure 6E. By contrast, when the particle is at the vesicle bottom, the local membrane slip velocity increases up to $v_{slip}^{bottom}\approx \eta_{m}2\pi f_{p}R_{p}$, generating a shear stress across the gap, that enables vesicle translation (slip). In good agreement with this scenario, we observe that *v*_*v*_ increases linearly with *v*_*p*_ (*v*_*p*_∝*f*_*p*_), as shown by the gray circles and cyan squares in Figure 6E.

This behavior contrasts sharply with that of vesicles with solid‐like membranes, which translate at constant velocity regardless of particle position due to the rigid‐body rotation of the vesicle membrane (red line, Figure 6D).^[^ ^33^ ^]^ Moreover, the translational velocity of vesicles with fluid‐like membranes when the particle is at the bottom is slightly lower than that of vesicles with solid‐like membranes, as shown by the symbols lying just below the red line in Figure 6E. This reduced velocity reflects the dissipative nature of fluid membranes, as they can deform and flow locally, allowing part of the stress to dissipate without contributing to the displacement of the vesicle as a whole.

## 3 Conclusion

Our results highlight the critical role of membrane mechanics in mediating force transduction from localized internal stresses—generated by the roto‐translation of an encapsulated particle—to whole‐vesicle motion. While solid‐like membranes transmit particle‐generated stresses efficiently to the vesicle center of mass through rigid‐body rotation, fluid membranes deform and flow locally, dissipating part of the stress. This leads to stick‐slip vesicle propulsion. Beyond regulating stress transmission, the fluid membrane also shapes the dynamic interplay between particle motion and vesicle deformation, which feeds back on the translation dynamics of the vesicle. The motion of the encapsulated particle is strongly influenced by the presence of boundaries outside the vesicle. Moreover, the particle is slowed down by the larger separation from the membrane imposed by repulsive forces arising from membrane shape fluctuations. Simultaneously, the fluid membrane deforms in response to the stresses exerted by the encapsulated particle, redistributing tension and accommodating excess area through local curvature changes. In phase‐separated membranes, line tension at the domain boundaries further influences motion by deflecting the particle from the direction imposed by the external force and gradually reorienting the domains. When the forces exerted by the particle overcome line tension, the initial Janus morphology evolves into a belt‐like configuration, which acts as a guiding track to stabilize the particle trajectory along the vesicle equator. Together, these observations show that the membrane is an active mechanical component that stores, dissipates and redirects internal stresses, reshaping itself in response to persistent active forces. This dynamic coupling between particle motion and membrane remodeling may help explain how force transduction and structural organization are co‐regulated in biological systems, where internal motors operate within deformable, fluid membranes.

## 4 Experimental Section

### Chemicals

All lipids were purchased from Avanti Polar Lipids. PEG, PVA, hexane, chloroform, glucose, sucrose and trimethoxy(octadecyl)silane were purchased from Aldrich. BSA was purchased from Biowest. Epoxi was puchased from RS Components. Acetone, isopropanol and ethanol purchased from VWR.

### Microfluidic Production of Lipid Vesicles Containing Ferromagnetic Particles

To form double emulsion drops with ultrathin oil shells, the glass‐capillary microfluidic device described in ref. [35] was used. Briefly, two tapered capillaries with an outer diameter (OD) of 1 mm (World Precision Instruments, WPI) were positioned coaxially within a square capillary with an inner dimension (ID) of 1 mm (VitroCom, CM Scientific). The tips of the cylindrical capillaries were facing each other within the square capillary and positioned 60μm apart. The tip diameter of the injection capillary was 60μm and that of the collection capillary was 120μm. The injection capillary was coated with trimethoxy(octadecyl)silane prior to device assembly to render its walls hydrophobic. An additional, smaller capillary, stretched with a flame to fit in the injection capillary, was inserted into it to inject the inner water phase, the aqueous suspension of ferromagnetic particles (nominal radius, *R*_*p*_ = 4 µm, carboxylic acid coating, CFM‐80‐5, Spherotech, Inc.) at a concentration of 1.5 mg mL^−1^. The middle oil phase was injected through the injection capillary. The outer water phase was injected through the interstices between the injection and the square capillary. The interstices of the collection and square capillaries were sealed with epoxy. As the inner water phase and middle oil phase flow through the injection capillary, they create a sequence of W/O single emulsion droplets. When these droplets reach the tip of the injection capillary, they encounter the continuous water phase, resulting in an alternating formation of O/W single and W/O/W double emulsion droplets with ultrathin oil shells. These droplets were collected in a collection medium adjusted to match the osmolarity of the inner water phase, 100 mM sucrose or 220 mM glucose, depending on the internal vesicle composition. Upon collection, single and double emulsions were easily separated due to their density differences, with single emulsions floating and double emulsions sinking to the bottom of the collection chamber. Flow rate‐controlled pumps (New Era Pump Systems, Inc.) were used to inject each phase into the device. Typical flow rates of 1000μL h^−1^ for the inner and middle phases, and 7000μL h^−1^ for the outer phase, were applied to ensure operation in the dripping regime.

Double emulsion drops spontaneously transform into vesicles in the collection chamber. The ultrathin thickness and chemical nature of the oil shell of the emulsions was critical for successful assembly of the lipid bilayer. Lipids are dissolved to a final concentration of 5 mg mL^−1^ in a mixture of 36 vol% chloroform and 64 vol% hexane. Upon evaporation of chloroform, which is more volatile and a better solvent for lipids than hexane, lipid adsorption at the two O/W interfaces of the double emulsion drop is promoted, while the shell thickness is reduced. Excess lipids in the hexane‐rich solvent mixture tend to aggregate inducing a depletion attraction between both monolayers and ultimately leading to the assembly of the lipid membrane through solvent dewetting.

### BSA‐Coated Substrate Preparation

Cover slides (24 × 50 mm, VWR) were first cleaned with soap, Milli‐Q water (Millipore, Merck), ethanol, isopropanol, and acetone before coating. After cleaning, 100μL of a freshly prepared 10%wt Bovine Serum Albumin (BSA) solution was applied to the slide and incubated for 15 min. The excess BSA was then removed by gently rinsing the slide with Milli‐Q water, and any remaining water was allowed to evaporate before the slides were used. The observation chamber was made with the BSA‐coated cover slide and a cover slip on top in a sandwich configuration with a separation distance of ≈300  µm.

### Magnetic Set‐Up and Video‐Microscopy

The magnetic set‐up consists of three orthogonal pairs of Helmholtz coils aligned along the *x*, *y*, and *z* axes coupled to an inverted bright‐field and epifluorescence optical microscope (Nikon ECLIPSE TS2R). Using a custom MATLAB code and a digital‐to‐analog converter DAQ (Measurement Computing USB‐1208HS), a sinusoidal signal, phase‐shifted by π/2 was sent to two of the coils. The choice of which coil pairs receive the signals depends on the desired axis of rotation for the applied magnetic field. To obtain a magnetic field strength of 10 mt, the signals were amplified with 500 W amplifiers (LD Systems). An oscilloscope (Tektronix TDS 2014B) was used to monitor the voltage sent to the coils. For both bright‐field and fluorescence imaging, a CCD camera (Grasshopper3 USB3, FLIR) with a 40× air objective (NA = 0.6), Nikon S PLAN FLUOR, was used. For fluorescent images, a 525 nm filter was used. The typical frame rate for bright‐field images was 10 fps, except during flicker experiments, where the frame rate is increased to 35 fps. Fluorescence images were captured at 1.53 fps. When a larger field of view was required, a 0.55× (Nikon) reduction lens was employed.

### Particle Tracking

Image analysis was performed using a custom MATLAB script adapted from the particle tracking algorithm originally developed by John Crocker and Eric Weeks.^[^ ^49^ ^]^ This method enables the extraction of 2D trajectories by identifying the center‐of‐mass positions of both the particle and the vesicle. While direct measurement of the particle's *z*‐position was not possible, variations in its focus provide qualitative information about its out‐of‐plane motion, suggesting it follows circular paths near the vesicle equator.

### Flickering Experiments

To obtain the fluctuation spectrum of the lipid vesicles, bright‐field images at the midplane were recorded at a frame rate of 35 fps. A 40× air objective with NA = 0.6 was used. At any given time, the contour of a fluctuating vesicle could be decomposed in a series of Fourier modes, *n*, where: $r\left(\theta \right)=R_{v}\left(1+\sum_{n=1}^{\infty }a_{n}\cos\left(n\theta \right)+b_{n}\sin\left(n\theta \right)\right)$.^[^ ^50^ ^]^ The fluctuation spectrum, 〈|ξ_*n*_|^2^〉, was determined by time‐averaging of the quadratic fluctuation amplitudes.

(1) $$\left\langle \right|\xi_{n}{|}^{2}\rangle =\frac{\pi {\left\langle R_{v}\right\rangle }^{3}}{2}\left[\langle \right|c_{n}{|}^{2}\rangle -\langle |c_{n}{\left|\right\rangle }^{2}]$$

where $|c_{n}|=\sqrt{a_{n}^{2}+b_{n}^{2}}$. Following Ref. [50], the vesicle contour was detected, compute *a*_*n*_, *b*_*n*_, and *c*_*n*_ along with their corresponding errors, and apply a correction for the finite spatial resolution of the images to account for pixelation‐induced background noise. The fluctuating spectrum relates to the membrane tension, σ, and bending κ in planar membranes through the equipartition theorem^[^ ^11^ ^]^ yielding:

(2) $$\left\langle \right|\xi_{q}{|}^{2}\rangle =\frac{k_{B}T}{\left(\sigma q_{⊥}^{2}+\kappa q_{⊥}^{4}\right)}$$

where $q_{⊥}=\sqrt{q_{x}^{2}+q_{y}^{2}}$. For vesicles, only the midplane is accessible and equation (2) transforms to:^[^ ^50^ ^]^

(3) $$\langle {\left|\xi (q_{x},y=0)\right|}^{2}\rangle =\frac{k_{B}T}{2\sigma }\left[\frac{1}{q_{x}}-\frac{1}{\sqrt{\frac{\sigma }{\kappa }+q_{x}^{2}}}\right]$$

Due to the limited integration time of the camera used, τ = 30ms, equation (3) was no longer valid because fluctuations with a shorter life‐time than τ were not correctly fitted. Therefore, for the fitting performed in Figure 3, the time corrected fluctuation spectrum, proposed in^[^ ^50^ ^]^, was used:

(4) $$\begin{array}{cc} \langle {\left|\xi (q_{x},y=0)\right|}^{2}\rangle & =\frac{1}{\pi }\int_{-\infty }^{\infty }\frac{k_{B}T}{4\eta q_{⊥}}\tau_{m}\frac{\tau_{m}^{2}}{\tau^{2}}\\ & \;\times \left[\frac{\tau }{\tau_{m}}+\exp\left(-\frac{\tau }{\tau_{m}}\right)-1\right]\;dq_{y} \end{array}$$

where, η is the viscosity of the solvent and τ_*m*_ is the fluctuation life‐time:

(5) $$\tau_{m}^{-1}=\left(\frac{1}{4\eta q_{⊥}}\right)\left(\sigma q_{⊥}^{2}+\kappa q_{⊥}^{4}\right)$$

### Statistical Analysis

Three vesicle compositions were analyzed: DOPC, DOPC/DPPC/Chol (3:3:2), and PEG‐PLA drawn from the previous study,^[^ ^33^ ^]^ all produced using microfluidic techniques. For DOPC vesicles, *N* = 3 independent production batches were carried out, and *n* = 26 vesicles were analyzed. For DPPC/DOPC/Chol (3:3:2), *N* = 6 batches were conducted, and *n* = 57 vesicles were analyzed. For PEG‐PLA (from^[^ ^33^ ^]^), *N* = 2 batches comprising *n* = 42 vesicles were reported. Each independent batch corresponds to a separate microfluidic production performed on a different day.

Each DOPC and PEG‐PLA vesicle was tested at four particle rotation frequencies (*f*_*p*_ = 1, 2, 5 and 10 Hz). DPPC/DOPC/Chol vesicles were tested at a single frequency per vesicle, given their strong frequency dependence.

Unless stated otherwise, results were reported as mean ± standard deviation (SD) of vesicle and particle velocities, computed over at least four complete loops of the encapsulated particle along the equatorial plane for each vesicle. No formal hypothesis testing was conducted; analyses focus on descriptive statistics with replication across independent production batches and vesicles. For flickering spectroscopy, each fluctuation spectrum was computed from at least *m* = 2000 contours per measurement to ensure statistical convergence of the mode amplitudes.

## Author Contributions

J.L.A. and L.R.A. contributed to conceptualization, investigation, resources, writing ‐ review and editing, supervision, funding acquisition, while P.M. carried out investigation, formal analysis, writing ‐ original draft, and A.E.O provided supporting investigation.

## Conflict of Interest

The authors declare no conflicts of interest.

## Supporting information

Supporting Information

Supplemental Movie 1

Supplemental Movie 2

Supplemental Movie 3

Supplemental Movie 4

Supplemental Movie 5

Supplemental Movie 6

Supplemental Movie 7

## Data Availability

The data that support the findings of this study are available from the corresponding author upon reasonable request.
