# Supplementary material for: Membrane‐Mediated Force Transduction Drives Stick‐Slip Motion of Lipid Vesicles
Source: Adv Sci (Weinh). 2026 Jan 26;13(11):e17219. doi: 10.1002/advs.202517219 (PMC12931262; doi:10.1002/advs.202517219)
Supplement: Supplementary file 1 — Supporting Information [file ADVS-13-e17219-s006.pdf]

Membrane-Mediated Force Transduction Drives Stick-Slip Motion of Lipid Vesicles

Paula Magrinya[a], Arin Escobar Ortiz[a], Juan L. Aragones[a],* and Laura R. Arriaga[a]†

[a] *Department of Theoretical Condensed Matter Physics,*

Condensed Matter Physics Center (IFIMAC) and Instituto Nicolás Cabrera,

Universidad Autonoma de Madrid, 28049, Madrid, Spain

* juan.aragones@uam.es

† laura.rodriguezarriga@uam.es

Electroformation of GUVs

To produce GUVs without PVA in the inner aqueous phase, we use the standard electroformation method [?]. We prepare a stock solution containing 37.4 mol% DOPC, 37.4 mol% DPPC, 25 mol% cholesterol, and 0.2 mol% DHPE-Rh at a final concentration of 0.5 mg/mL in chloroform. We deposit 250 μL of this lipid solution onto the conductive surfaces of two indium tin oxide (ITO, 15-25 Ω/sq, Aldrich) glass slides and allow the solvent to evaporate completely. We then assemble a closed chamber by sandwiching the ITO slides together, using Vitrex® as both a spacer and chamber seal. The chamber is filled with an aqueous solution of magnetic particles (Dynabeads® M-270 Carboxylic Acid, R_p = 1.35 μm) at 1.5 mg/mL in 200 mM sucrose. An AC electric field (2 V amplitude, 10 Hz) is applied across the ITO slides for 2 hours at 40 °C using a function generator (JDS6600). After electroformation, we disperse the resulting GUV suspension into an excess volume of 200 mM glucose solution.

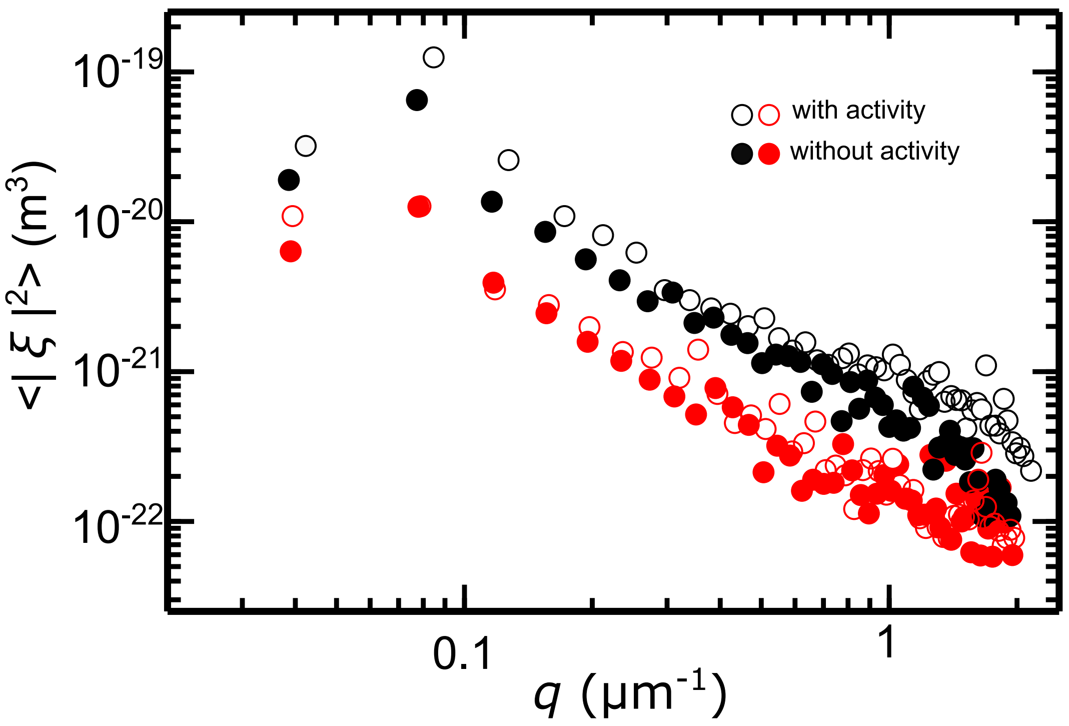

FIG. S1: Difference in the fluctuation spectrum in a vesicle encapsulating a ferromagnetic particle. Empty circles show when the particle is actuated and filled circles when it is not. Different colors represent different membrane tension. Data is presented as mean ± SD for over 2000 contours.

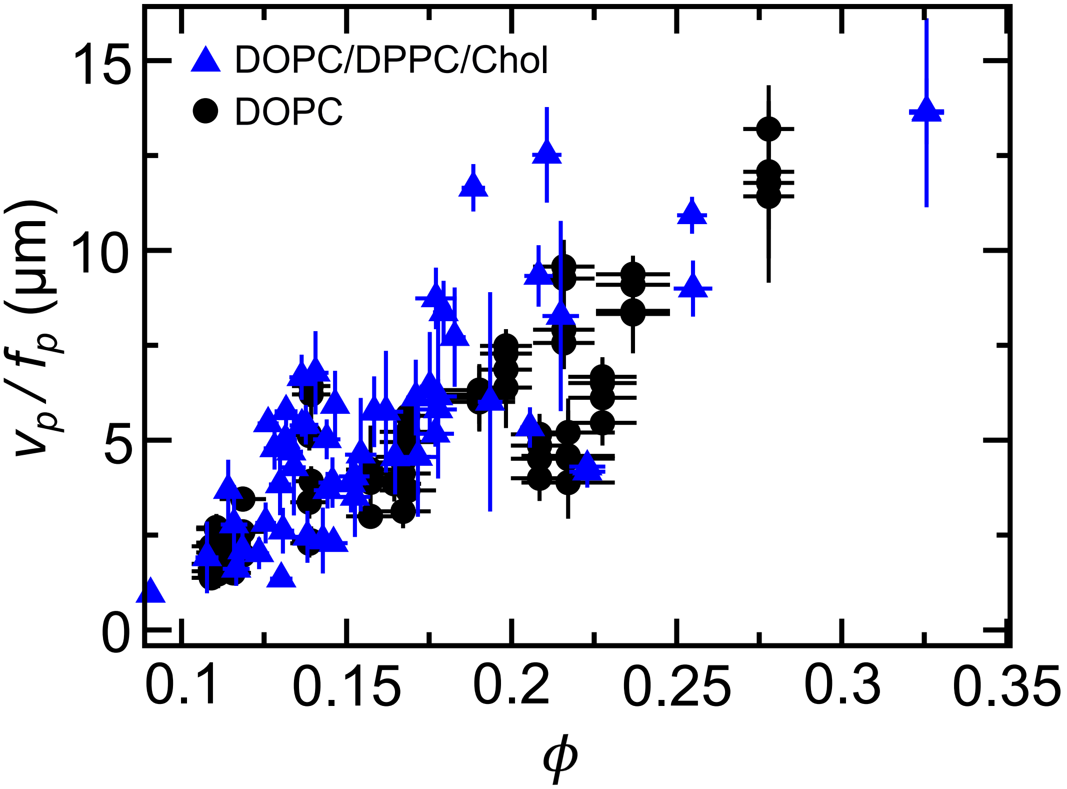

FIG. S2: Distance traveled in each particle's rotation, v_p/f_p, as a function of degree of confinement, ϕ. Particle is either encapsulated in a vesicle composed of either just DOPC or a lipid mixture of DOPC/DPP/Chol. Data is presented as mean ± SD with n=57 for DOPC/DPPC/Chol and n=19 for DOPC

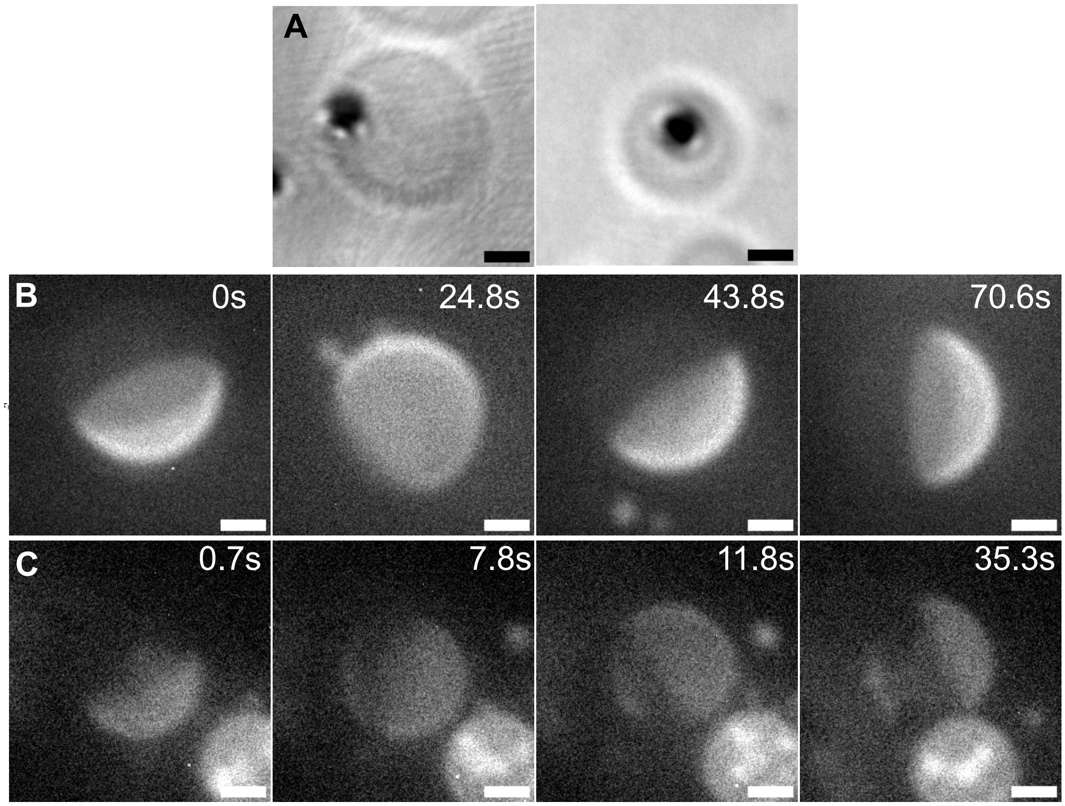

FIG. S3: (A) Bright field microscope images of electroformed lipid vesicles encapsulating a magnetic particle. (B-C) Epi-fluorescence microscope images of a phase-separated vesicle, with the L_d phase labeled in white. Particle rotating around the x-axis induces: (B) domain alignment perpendicular to the x-axis when $\tau_h < \tau_\lambda$ or (C) a one-phase belt when $\tau_h > \tau_\lambda$. Scale bar 5 μm.

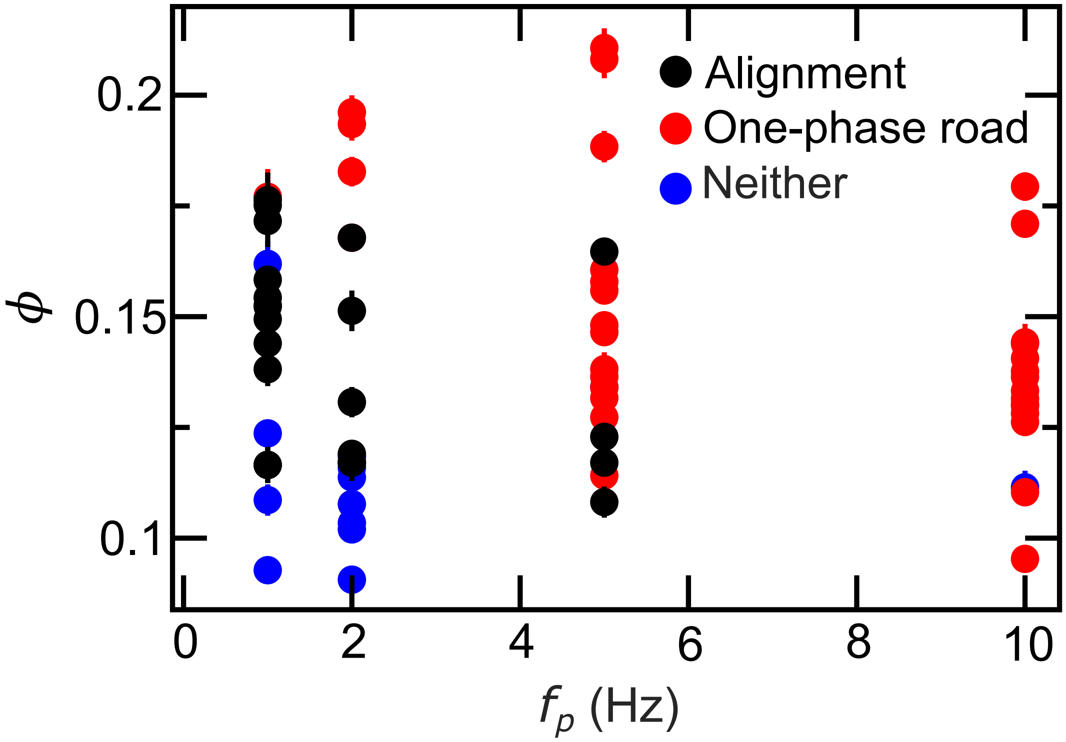

FIG. S4: Phase diagram illustrating the behavior of phase-separated vesicles, showing three distinct regions: Domain Alignment, One-Phase road, and neither of them. The diagram is plotted as a function of the degree of confinement and encapsulated particle frequency. Data is presented as mean ± SD with n=70.

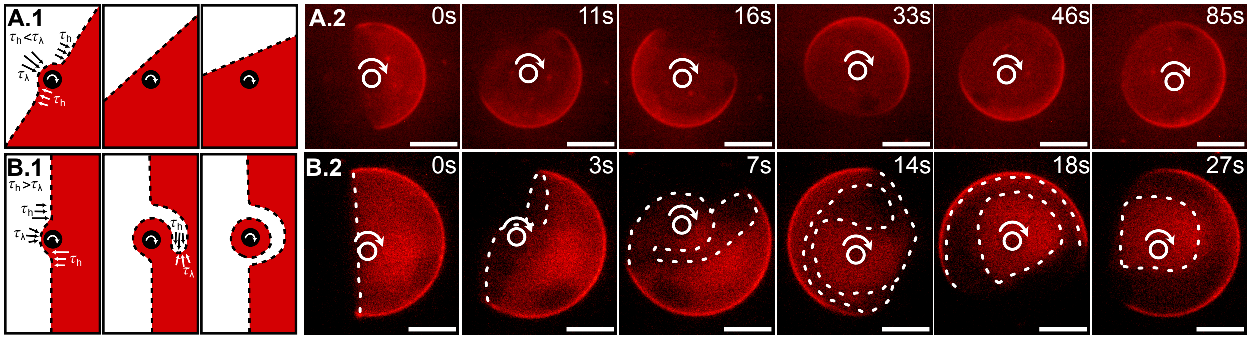

FIG. S5: Schematic illustration (left) and Epi-fluorescence microscope images (right) of a phase-separated vesicle, with the L_d phase labeled in red; and the encapsulated particle, slightly visible as a dark spot highlighted as a white circle for visualization purposes. Particle spinning around the z-axis induces: (A) domain alignment perpendicular to the z-axis when $f_p = 5$ Hz ($\tau_h < \tau_\lambda$) or (B) a one-phase belt at the equator when $f_p = 10$ Hz ($\tau_h > \tau_\lambda$). Scale bar 25 µm.

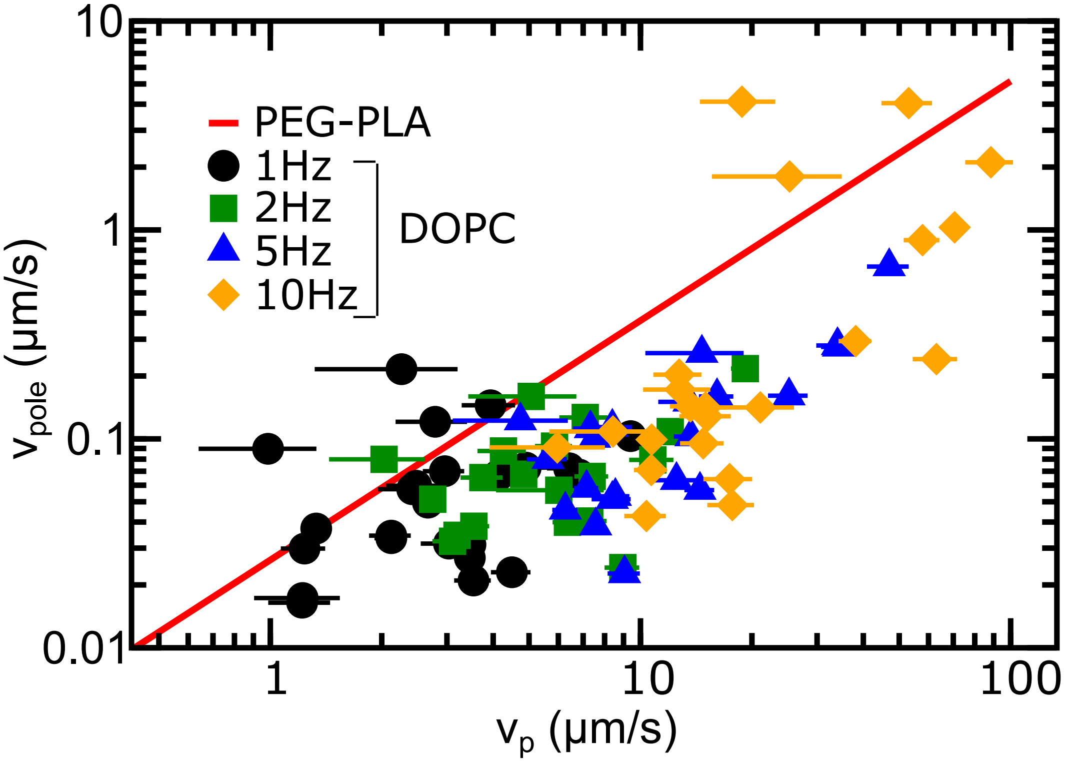

FIG. S6: Drift particle velocity towards one of the vesicles poles, v_{pole} as a function of particles velocity v_p for particles actuated at different frequencies encapsulated inside a DOPC or a PEG-PLA vesicle. Data is presented as mean ± SD with n=24.

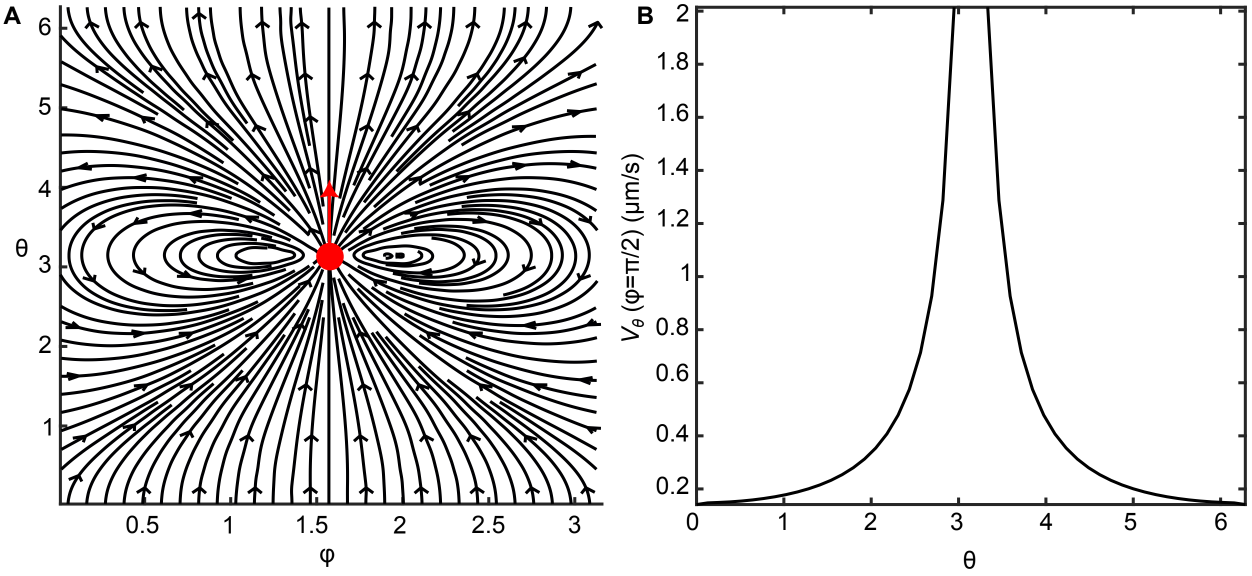

FIG. S7: A) Streamlines generated by a point force in the a vesicle membrane. B) Membrane velocity along the $\varphi = \pi/2$ streamline. The further from the point force the smaller the velocity. Here, $R_v = 35.5\,\mu\text{m}$, $F = 0.25$ pN, $\eta_m = 4 \cdot 10^{-9}$Pa·s·m and $\eta_{3D} = 10^{-3}$ Pa·s. We have used the model described in [**?**].

Movie SM1. Behavior of a rotating particle encapsulated within a lipid vesicle (DOPC). Particle rotates with $f_p = 2$ Hz and the degree of confinement is $\phi = 0.22$. The behavior of a free particle can also be seen.

Movie SM2. Behavior of a rotating particle encapsulated within a phase-separated lipid vesicle (DOPC/DPPC/Chol). The particle performs diagonal movements when it encounters the domain interface, which can be seen due to the slight budding of the vesicle. Particle rotates with $f_p = 1$ Hz and the degree of confinement is $\phi = 0.14$.

Movie SM3. Alignment of domains in roller mode. Particle rotates around the **x**-axis and domains tilt until their boundary is perpendicular to the axis of rotation. $f_p = 1$ Hz and $\phi = 0.15$.

Movie SM4. Domains rupture in roller mode. Particle forms a $L_{\mathbf{o}}$ road and performs circular loops within the road. $f_p = 5$ Hz and $\phi = 0.16$.

Movie SM5. Domains rupture in roller mode. Particle forms a $L_{\mathbf{d}}$ road and performs circular loops within the road. $f_p = 10$ Hz and $\phi = 0.14$.

Movie SM6. Behavior of a rotating particle encapsulated within a lipid vesicle (DOPC). The particle deviates from the equator of the vesicle and spirals towards one of the vesicle poles but fails to reach it. $f_p = 5$ Hz and $\phi = 0.12$.

Movie SM7. Non uniform rotation of the vesicle's membrane. Defects (tagged in blue and black) have different rotational frequencies depending on the particle's position (tagged in magenta). $f_p = 5$ Hz and $\phi = 0.18$.
